# Estimating Income-Related Health Inequalities Associated with Tobacco and Alcohol Consumption in Namibia

**DOI:** 10.3390/ijerph20021062

**Published:** 2023-01-06

**Authors:** Martha Tangeni Nghipandulwa, Alfred Kechia Mukong

**Affiliations:** Department of Economics, University of Namibia, Windhoek 13301, Namibia

**Keywords:** income-related health inequality, tobacco and alcohol, concentration curve, corrected concentration index, Namibia

## Abstract

Disparities in resources and access to material opportunities are important determinants of income-related health inequality. This paper hypothesises that the gradient of the inequality in health between the poor and the rich is likely to depend on differences in lifestyle practices including tobacco use and alcohol abuse. Using the 2015/16 Namibia Household Income and Expenditure Survey and the Erreygers corrected concentration index, we estimate the effect of tobacco and alcohol use on income-related health inequalities. A decomposition technique was used to estimate the separate and joint contribution of tobacco and alcohol use to income-related health inequalities. The results indicate that tobacco use widens the income-related health inequality gap while alcohol consumption reduces health disparities. The simultaneous consumption of these goods has a stronger multiplicative effect on income-related health inequality. For instance, the simultaneous consumption of both goods contribute up to 1.03% of the inequality in health while tobacco use alone contributed only 0.6%. While policy options for each of these goods could be essential in reducing inequalities in health, there is a need to advocate additional measures that could simultaneously control the consumption of both goods.

## 1. Introduction

While reducing premature mortality from non-communicable diseases (NCDs) is now on the post-2015 development agenda, it is estimated that by 2030, deaths from NCDs will be five times higher than deaths from communicable diseases in low- and middle-income countries [1]. The rapid acceleration of NCDs is attributed to lifestyle changes, including smoking and harmful alcohol use. Tobacco use and alcohol abuse are among the major causes of NCDs and premature mortality [2,3] and account for over 8 million and 3.3 million deaths, respectively, each year [4,5]. While many countries are experiencing a significant improvement in population health status [6], socioeconomic-related health inequality remains a public health challenge for both developed and developing countries [7,8,9,10]. Health outcomes vary with socioeconomic status, but the gradient of socioeconomic-related health inequality can be exacerbated by unhealthy lifestyle practices including unhealthy diet, tobacco use, alcohol abuse and physical inactivity [9,11,12].

Evidence suggests that socioeconomic-related inequalities in health are attributed to disparities in resources and access to material opportunities such as employment, education, nutrition and household assets [13,14,15]. In addition, unhealthy practices have been shown to have negative health effects and, if concentrated among the socioeconomically disadvantaged or vulnerable individuals, the inequality gap will widen [9,11,16,17]. There is vast evidence on the economic burden of tobacco and alcohol consumption [18,19,20], the health effect of tobacco and alcohol consumption [21,22] and the economic burden of smoking and alcohol-related diseases [23,24]. However, there is growing but limited evidence on the contribution of tobacco use and alcohol abuse to income-related inequalities in health. This is particularly the case in many African countries where data availability has hampered research in this area.

Some studies, although few, have demonstrated that tobacco use and alcohol abuse contribute positively to socioeconomic-related inequalities in health, suggesting that the health effect of these goods is higher among the poor [9,11]. However, there is a scarcity of studies estimating the effect and contribution of these goods on socioeconomic health inequality in the African context. One study using data from South Africa concluded that income-related health inequality is higher for alcohol-consuming than cigarette-smoking individuals and the health effects were more severe when the two goods are consumed together [9]. While such studies have been important for the formulation of anti-smoking and alcohol policies, the focus has been on self-assessed health (SAH), a subjective evaluation of health, rather than more objective measures [9,11,25]. Mukong et al. [9] further note that while alcohol and tobacco alone are strongly related to the risk of ill health, simultaneous exposure to these goods had a stronger multiplicative effect on health inequality. A review of the literature indicates that evidence of the joint effect of these substances on income-related health inequality remains scarce. There is, therefore, a need to unpack the joint effects of tobacco and alcohol on income-related health inequality, using more objectively related health measures. This paper contributes to the growing literature by analysing the independent and joint effects of tobacco and alcohol use on income-related health inequality in Namibia. The paper also considers health outcomes that are directly associated with tobacco and alcohol consumption.

Recent estimates suggest that smoking prevalence in Namibia is 18.6%, a decline from 21.6% in 2015 [26]. However, smoking prevalence is expected to increase to 26.9% by 2025 if tobacco control efforts are not intensified [27]. The per capita alcohol consumption in 2016 was estimated at around 9.8 litres per day and is expected to decline to 8.5 litres per day by 2025 if appropriate policies are implemented within the country [4]. This is still worrisome, as 8.5 litres per day is far above the current African and world averages of 6.3 litres and 6.4 litres per day, respectively. In Namibia, smoking prevalence and alcohol use are disproportionately higher among the rich than among the poor [28] and among men than women [29]. The country ratified the World Health Organisation’s Framework Convention on Tobacco Control in 2005, yet implementation remains a challenge due to the lack of capacity to enforce the regulations [30]. Thus, more evidence is required to strengthen public health policy in relation to the consumption of tobacco and alcohol. While several studies have identified the determinants and the health effects of these goods [28] no study has estimated the associated income-related health inequality.

We address this critical evidence gap by examining the independent and joint contribution of tobacco and alcohol to income-related health inequality in Namibia. We hypothesise that differences in tobacco and alcohol use between the poor and the rich are likely to affect the gradient of income-related health inequality. We used data extracted from the Namibia Household Income and Expenditure Survey to test this hypothesis. We control for individual socioeconomic variables that are known empirically or theoretically to be associated with income-related health inequality [9,11,25]. An understanding of these relationships is key to the implementation of context-specific tobacco and alcohol control policies in Namibia.

## 2. Materials and Methods

### 2.1. Data and Key Variables

We used data extracted from the Namibia Household Income and Expenditure Survey (NHIES). The data are publicly available from the Namibia Statistics Agency Website (Central Data Catalogue—Namibia Statistics Agency (https://nsa.org.na/page/central-data-catalogue (accessed on 21 November 2022))). The survey was conducted by the Namibia Statistics Agency (NSA) between April 2015 and March 2016. The 2015/16 NHIES is the most recent nationally representative survey, with data from 10,368 households in the 14 regions of the country with detailed information on the disease profile, the socioeconomic status and the tobacco and alcohol consumption behaviour of individuals.

A two-stage stratified cluster sampling procedure was used and was based on the 2011 Population and Housing Census Enumeration Areas (EAs). In the first stage, 864 clusters were selected and in the second stage a total sample of 10,368 households was selected, although only 10,090 households were successfully interviewed. The data were collected over twelve months consisting of thirteen survey rounds to account for seasonal changes that may affect household expenditure or income patterns. It provides information on several tobacco and alcohol-related diseases including diabetes, high blood pressure, joint inflammation, cancer, heart disease, respiratory disease, chronic kidney disease, anaemia, and psychological and other chronic diseases. It also collected information on individual tobacco and alcohol consumption behaviour as well as household income and expenditure.

### 2.2. Measurement of Key Variables

Households differ in size and demographic structures and thus using aggregate household income as a measure may be misleading. We use both household per capita income and household per capita income by adult equivalent as measures of income. Household per capita income assumes that household expenditure on a child and an adult is the same. The literature has consistently advocated the need to control household demographic composition when comparing income across households. This approach assumes that household expenditure on a child is generally smaller than expenditure on an adult [31,32,33]. In this paper, household income is converted into household income per “equivalent adult” by assuming that household monthly expenditure on a child is smaller than expenditure on an additional adult.

The household income per adult equivalent assumes that if E is a measure of the household expenditure index, then E is likely to depend on household size and age composition of household members. The unadjusted household income per adult equivalent is AE=X/E, where AE is the adult equivalent household income and X the unadjusted household income. While there are different formulations for E, the double parameter class of equivalence scales as formulated by Cutler and Katz [34] in Equation (1) is commonly used.

(1) $$E={\left(N_{A}+cN_{C}\right)}^{\theta }$$

where $N_{A}$ is the number of adults and $N_{C}$ is the number of children; c is a parameter for expenditure on a child relative to that on an adult and θ measures overall economies of scales within the household. A child is equivalent to an adult when *c* = 1 [32]. However, the values of c and θ are mostly between 0 and 1 and may differ between countries. While the values of c and θ have not been established for Namibia, we adopt the South African values of c=0.5 and θ=0.9 as proposed by Deaton in 1993 [31]. Using a variety of combinations of c and θ for meaningful comparison, Woolard & Leibrandt [33] found no significant difference from the benchmarked values of c=0.5 and θ=0.9. The adoption of these values for Namibia is based on the assumption that Namibia and South Africa are both upper middle-income countries, belonging to the same regional blocs, and Namibia was colonised by the apartheid South African government until independence in 1990.

This paper made use of tobacco and alcohol-related health outcomes to estimate income-related health inequalities associated with tobacco and alcohol consumption. The selection of these health outcomes is guided by existing literature and data availability. The health measures are binary outcomes equivalent to 1 if the respondent reported to have been diagnosed with a particular disease and zero otherwise. For a more generic measure, we constructed a composite health index using the Min-Max rescaling transformation procedure. In the Min-Max rescaling transformation method, each variable is decomposed into an identical range between 0 and 1. A score of 0 is the worst rank for a specific indicator and a score of 1 is the best. In this paper, the score is 0 if an individual is not suffering from a specific chronic disease and 1 otherwise. All other values are then scaled between the minimum and maximum values. According to Yoon [35], the scaling procedure ultimately subtracts the minimum value ($X_{min}$) and divides it by the observed difference between the maximum value ($X_{max}$) and the minimum value ($X_{min}$) as follows:

(2) $$C_{i}=\frac{X_{i}-X_{min}}{X_{max}-X_{min}}$$

where $X_{i}$ is equivalent to 1 if diagnosed with a particular chronic disease and 0 otherwise. The approach has been used by several scholars to aggregate variables and create composite scores [36,37,38,39]. It has also been used to compute human development indices. The indices generated were standardised and the standardised indices are between 0 and 1. There are 12 indicators of chronic diseases used in computing the health index and thus, the standardisation was obtained by calculating the average values of the different diseases.

(3) $$HI=\frac{\sum_{i=1}^{n}IndexA_{i}}{n}$$

where $IndexA_{i}$ are the different health indicators that make up the health index and n is the number of chronic diseases used (12 in our case). However, given that all health indicators are coded as 0 and 1, the health index simply follows an additive process. The composite index ranges from 0 to 1 with high values denoting a higher prevalence rate of related diseases.

### 2.3. Analytical Method

#### The Concentration Index

The health concentration index (CI) was used to estimate the extent of income-related health inequality. The CI is a bivariate measure that has been extensively used to measure socioeconomic-related inequalities in health, by ranking related health outcomes to other variables including income and education as well as smoking and alcohol [10,11,40]. The concentration index ranges between −1 and +1, with positive values suggesting that the inequality is in favour of the rich, negative values suggest it favours the poor and 0 that the health of the population is evenly distributed among the rich and the poor [41]. That is, a value of −1 suggests that poor health is concentrated among the poor and +1 suggests that poor health is concentrated among the rich [40,42].

The standard CI has the potential of summarising the extent of inequality in a single measure, but it may not be a good measure for comparing health inequality between countries and over time, especially when health indicators are bounded [43]. When the health outcome is binary, the bounds of the CI depend on the mean (μ) of the variable and range between μ−1 and 1−μ. According to Wagstaff [43], the CI can be normalised using (1−μ). However, Erreygers [44] claims that this is an ad hoc procedure, suggesting the use of a corrected concentration index (CCI). The argument is that CCI satisfies level independence such that equal increments in the health of all individuals do not affect the value of the index. The health indicators used in this paper are binary and bounded in nature (between 0 and 1) suggesting the use of the Erreygers CCI specified as follows:

(4) $$CCI=\frac{4\mu }{d-e}\times C$$

where μ is the mean health status, *C* is the standard CI, *d* is the maximum level of health (1) and *e* is the minimum level of health status (0). The CCI only modifies the standard CI to satisfy the desired properties of a rank-dependent index but can be decomposed using the same decomposition technique. We estimated the contribution of tobacco and alcohol use on income-related health inequality by decomposing the CCI [45,46]. This requires the health variable to be first regressed as a function of its determinants.

(5) $$h_{i}=\alpha +\overset{n}{\underset{k=1}{\sum }}\beta_{ki}x_{ki}+\varepsilon_{i}$$

where $h_{i}$ is the health outcome of individual i, $x_{ki}$ is a set of covariates, including tobacco and alcohol consumption, *α* is the constant and $\varepsilon_{i}$ is the error term. The decomposed CCI is the weighted sum of the CI for each health covariate and can be re-written as:

(6) $$CCI=4⎡\overset{}{\underset{k}{\sum }}\left(\beta_{k}GC_{k}\right)+GC_{\varepsilon }⎤=4⎡\overset{}{\underset{k}{\sum }}\left(\left(\beta_{k}{\bar{x}}_{k}CI_{k}\right)\right)+GC\varepsilon ⎤$$

where ${\bar{x}}_{k}$ and $CI_{k}$ are the means of $x_{k}$ and CI respectively, $GC_{k}$ and $GC_{\varepsilon }$ are the generalised concentration indices for $x_{k}$ and the error term. This quantifies the contribution of tobacco and alcohol use to health disparities linked to income. The overall contribution to income-related health inequality is the product of three separate components, namely, the coefficient $\beta_{k}$; the prevalence of each variable given by its mean ${\bar{x}}_{k}$; and the distribution of the variable across income groups, given by the concentration index $CI_{k}$. The decomposition approach is one-dimensional and ignores the covariance between health and income [47], and gives little thought to identification strategies [48,49]. However, the Wagstaff et al. [45], decomposition is regarded as the most appropriate in the literature since structural equation modelling is data-demanding.

## 3. Results

### 3.1. The Concentration Curves

A concentration curve is used to illustrate income-related health inequality. It shows the cumulative share of the population that reported being diagnosed with a tobacco/alcohol-related disease against the cumulative population shares, ranked by household income. If the concentration curve coincides with the 45-degree line, it indicates that the prevalence rate of the disease is equally distributed across the income groups, implying a proportional distribution. However, if the disease profile is more than proportionately concentrated among the poor, the concentration curve would lie above the 45-degree line. On the contrary, if the disease profile was more than proportionately concentrated on the richer population, the concentration curve would lie below the line of equality [50].

**Figure 1:**
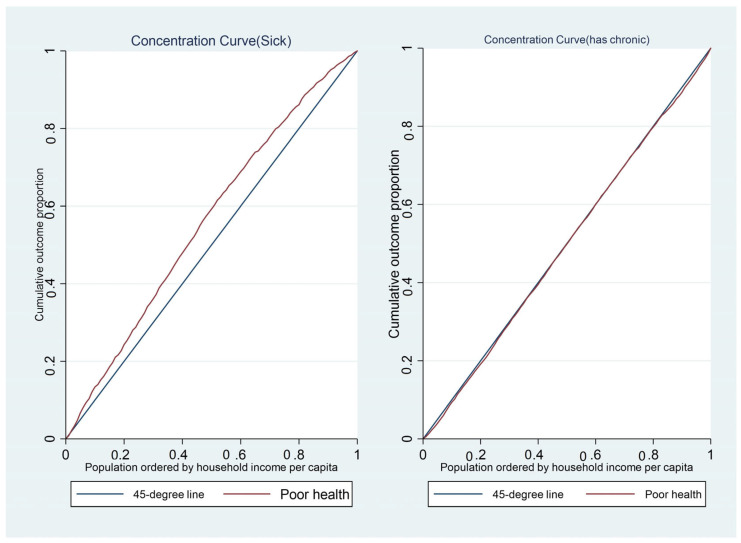
The concentration curves for being sick and for having a chronic disease.

**Figure 2:**
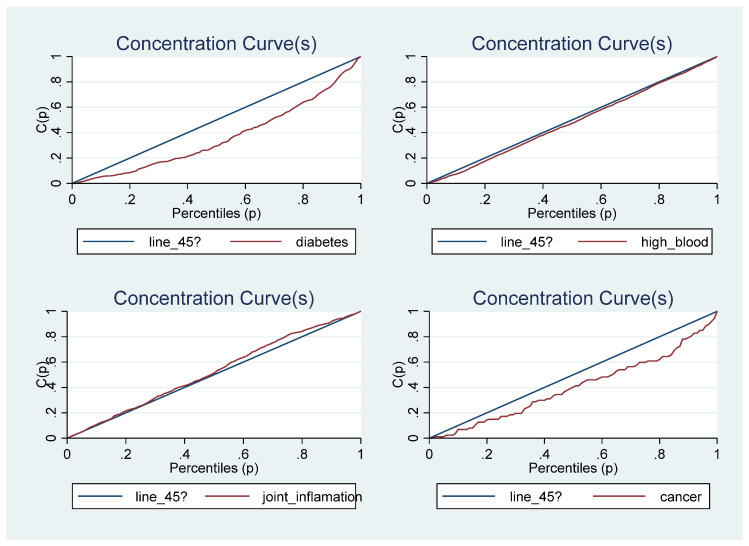
The Concentration Curves for some tobacco/alcohol-related chronic diseases.

**Figure 3:**
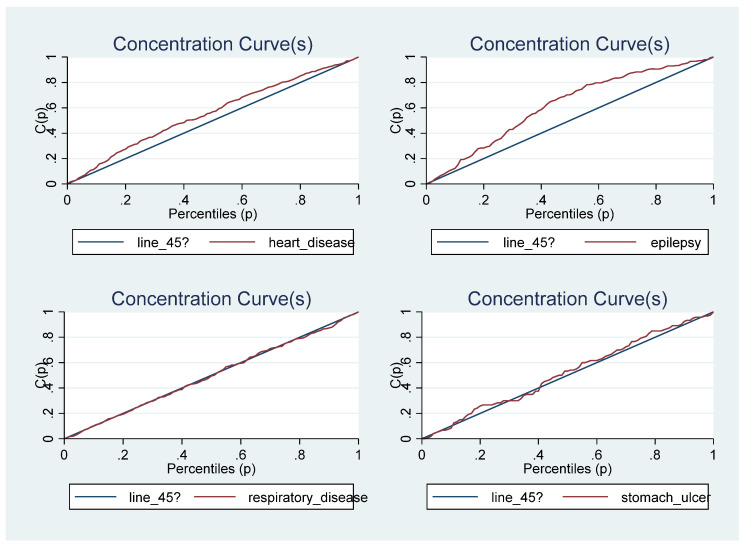
The Concentration Curves for some tobacco/alcohol-related chronic diseases.

**Figure 4:**
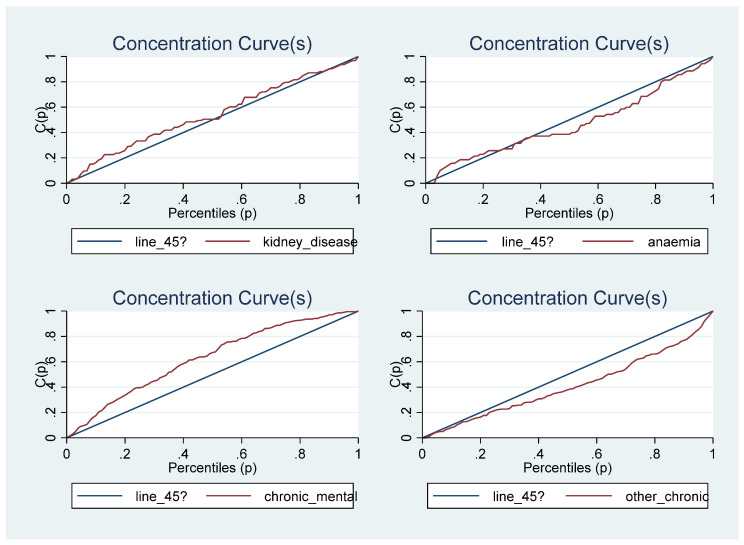
The Concentration Curves for some tobacco/alcohol-related chronic diseases.

Figure 1 shows the concentration curves for being sick and/or for being diagnosed with a chronic disease. The results suggest that income-related health inequalities were generally concentrated on the poor when being sick is the considered health outcome and the dominance cannot be determined when chronic disease is the health outcome. The concentration curves for the individual chronic diseases are presented in Figure 2, Figure 3 and Figure 4. While some of the diseases are concentrated among the poor (heart disease, epilepsy, stomach ulcer, kidney disease and chronic mental illness) the dominance of others cannot be determined (high blood pressure, joint inflammation and respiratory disease) and others are concentrated among the rich (diabetes, cancer, anaemia and other chronic diseases). This may suggest why the dominance of the concentration curve for being diagnosed with a chronic disease cannot be determined. The concentration curves in Figure 1 suggest that being sick is more pro-poor than having a chronic disease, given that the former generally lies somewhere above the line of equality while the latter curve mostly coincided with the line of equality for most parts of the income distribution.

The concentration curve is important in illustrating socioeconomic inequality at each point in the income distribution for the health outcomes of interest, but it cannot be used to quantify the magnitude of such socioeconomic inequality [51,52]. It is impossible to determine the dominance when the concentration curves cross each other. Thus, it is important to quantify the magnitude of socioeconomic inequality in each health outcome of interest with a summary index; this necessitates the estimation of the concentration index, especially for individual tobacco/alcohol-related diseases.

### 3.2. Descriptive Statistics

Table 1 presents the student t-test for differences in mean health outcomes by the tobacco and alcohol consuming status of individuals, offering interesting patterns in the distribution of chronic diseases between tobacco and alcohol consumers. A positive mean difference suggests that tobacco users and alcohol consumers are more likely to suffer from such a disease. The results depict that about 8% of the sample reported being sick 30 days before the survey while 20% were diagnosed with a chronic disease. Over 13% of the sample were diagnosed with high blood pressure, 2% with diabetes, joint inflammation, heart disease, and respiratory diseases; 1% with chronic psychology or mental health, epilepsy and other chronic diseases, and less than 1% with cancer, stomach ulcers or kidney disease.

The results show that the prevalence rate of these diseases is generally and significantly higher among smokers than non-smokers. For example, smokers are on average, significantly more likely to be sick and diagnosed with a chronic disease, joint inflammation, high blood pressure, heart disease, epilepsy, respiratory disease, stomach ulcer, kidney disease, chronic psychological and other chronic diseases than non-smokers. The results further suggest that alcohol users are more likely to be diagnosed with joint inflammation than their counterparts, but are significantly less likely to be diagnosed with a chronic disease, diabetes, high blood pressure, cancer and heart and respiratory disease. While these results seem surprising, it is important to note that the optimal level of alcohol consumption is not zero, since it has both positive and negative health effects. Mukong et al. [9] show that it is harmful and excessive alcohol use that contributes significantly to the prevalence of these diseases. While the effects of alcohol on health depend on the pattern and volume of alcohol consumed, we do not have sufficient information on alcohol consumption intensity to tease out this relationship.

### 3.3. The Distribution of Disease Burden by Household Income Quintile

In Table 2, we present summary statistics on the distribution of smoking prevalence, alcohol consumption and disease burden by income quintile. The results indicate that the prevalence rate of tobacco consumption is primarily higher among those in lower income quintiles while the alcohol prevalence rate is higher among individuals from affluent households. For instance, 24% of those in the poorest income quintile consume tobacco compared to 19% in the highest income quintile when household income per capita is used and 26% and 18%, respectively, when household income per capita adult equivalent is used. Over 18% of those in the poorest income quintile consume alcohol compared to 22% in the highest income quintile when household income per capita is used and 18% and 21%, respectively, when household income per capita is adult equivalent. This is consistent with evidence from South Africa [9].

It is evident from Table 2 that 24% of those in the poorest quintile reported being sick 30 days before the survey compared to 14% in the richest quintile, but the distribution of the prevalence rate of having a chronic disease varies significantly across the income quintiles. The prevalence of chronic diseases such as joint inflammation, heart disease, epilepsy, kidney disease, stomach ulcer and chronic mental health is higher among individuals in the poorest quintiles while cancer, anaemia, respiratory disease, high blood pressure and diabetes are more prevalent in the richest quintile.

### 3.4. The Corrected Concentration Indices for Income-Related Health Inequalities

Table 3 presents the corrected concentration indices for income-related, tobacco-related and alcohol-related health inequality for each health outcome. The corrected concentration indices (CCI) for both tobacco prevalence and consumption intensity are generally positive and significantly different from zero, suggesting that poor health is concentrated among tobacco users and even more among heavy users (see Columns 1 & 2 of Table 3 for comparison). The magnitude of the inequality is significantly higher when tobacco consumption intensity rather than tobacco prevalence is used. Tobacco-related health inequality ranges between −0.005 and 0.046 when tobacco consumption intensity is used and between −0.009 and 0.031 when tobacco prevalence is used. For example, tobacco-related inequality for being sick is 0.025 and having a chronic disease is 0.035 when tobacco prevalence is used, compared to 0.027 and 0.031, respectively, when tobacco consumption intensity is used.

Concerning alcohol-related health inequalities, the corrected concentration indices (CCI) are negative and significantly different from zero for having a chronic disease, diabetes and high blood pressure, suggesting that these diseases are concentrated among non-alcohol users. On the contrary, the CCI are positive and significantly different from zero for the sick, those with joint inflammation and chronic psychological health issues, suggesting that they are concentrated among alcohol users. The findings on alcohol suggest the need to investigate the CCI for alcohol consumption intensity since the health effects are supposed to vary depending on the volume and pattern of consumption rather than just the prevalence.

### 3.5. The Regression Estimates for Tobacco/Alcohol-Related Health Outcomes

The results in Table 4 present estimates of the effect of tobacco and alcohol use on related health outcomes. The results in columns (1) and (4) are probit marginal effect for being sick, columns (2) and (5) are probit marginal for having a chronic disease and columns (3) and (6) are OLS estimates of the effect of tobacco and alcohol on the health index. For results in column (1) to (3), an interaction term for tobacco and alcohol use is introduced to understand how having both smoking and drinking habits affect related health outcomes. The results show that having both habits significantly increases the probability of being sick from a related chronic disease. The results illustrate that consuming alcohol increases the probability of being sick by 0.8% points, of reporting a chronic disease by 2.5% points, and it increases the health index score by 0.01 units. Tobacco consumption has no significant effect on the probability of having a chronic disease and health index but significantly increases the probability of being sick by 2.1% points.

### 3.6. The Contribution of Smoking and Alcohol to Income-Related Inequalities

Table 5 summarises the percentage contributions of tobacco and alcohol consumption to the observed income-related health inequality. The contribution of each variable can be positive or negative, depending on the sign of its health effects and its distribution by income (shown by the sign of the *CCI*). A positive (negative) percentage contribution of each covariate implies that, ceteris paribus, income-related health inequality will be higher (lower) if the covariate is equally distributed across income groups, or the covariate has a zero-health elasticity. Tobacco accounts for 0.1% of all measured inequality in the health index, 0.06% for being sick and a maximum of 0.6% for inequality in the prevalence of chronic diseases.

Concerning individual health outcomes, tobacco use contributes positively to income-related inequality in being sick, being diagnosed with diabetes, joint inflammation, cancer, epilepsy, respiratory diseases, stomach ulcer, anaemia, chronic mental health and other chronic diseases. This suggests that tobacco consumption widens the income-related health inequalities gap for these diseases. However, tobacco consumption contributes negatively to inequality in high blood pressure, heart disease and kidney disease. Many of these findings are consistent with existing evidence that found a positive contribution of tobacco to income-related health inequalities [9,11]. Mukong et al. [9] use similar health indicators and found that cigarette smoking contributes between 3% and 8% to income-related inequality in health. Vallejo-Torres and Morris [11] use the EQ-5D (a generic measure of health status which is applicable to a wide range of health conditions and treatments and provides a descriptive profile that is reducible to a single index value for health status.) as a measure of health and found that smoking contributes up to 2.3% to income-related health inequalities. Thus, any policy options that reduce tobacco consumption are important in reducing the income-related inequality gaps for the associated diseases.

Contrary to tobacco use, alcohol consumption reduces (contributes negatively to) the income-related inequalities for many diseases, accounting for −1.26% of all measured inequality in the health index, −0.53% for being sick and −5.98% for inequality in the prevalence of chronic diseases. For individual health outcomes, alcohol consumption contributes negatively to income-related inequality in diabetes, joint inflammation, cancer, epilepsy, respiratory diseases, stomach ulcer, anaemia, chronic mental health, other chronic diseases, high blood pressure and heart disease but contributes positively to inequality in kidney disease. These findings are contrary to existing evidence that found a positive effect and contribution of alcohol consumption on related health inequalities [9,53]. The differences in results could be attributed to differences in the measures of alcohol used. For example, Mukong et al. [9] use alcohol intensity instead of alcohol prevalence.

Interestingly, the data allowed us to estimate the joint contribution of tobacco and alcohol consumption on income-related health inequality. Table 6 presents the joint contribution of tobacco and alcohol consumption to income-related health inequality. The analysis is limited to self-reported health (being sick within 30 days before the survey), having a chronic disease and the health index for chronic diseases. The simultaneous consumption of tobacco and alcohol is positive, accounting for 0.25% of all measured inequality in the health index, 0.13% for inequality in being sick and a maximum of 1.03% for inequality in being diagnosed with a chronic disease. The contributions from tobacco consumption alone are generally positive while those from alcohol consumption alone are negative. The outcomes are also consistent with the literature suggesting that while alcohol and tobacco alone are strongly related to the risk of ill health, simultaneous exposure had a strong multiplicative effect on health [9,54,55,56].

## 4. Discussion

Several studies have measured the contribution of tobacco and alcohol use to socioeconomic-related inequalities in health [9,11,40]. These studies consistently found that tobacco and alcohol abuse contribute positively to income-related health inequality. In the context of Africa, there is a dearth of research estimating the effect and contribution of these goods on socioeconomic-related health inequality. A review of the literature indicates that evidence of the joint effect of these goods on income-related health inequality remains scarce. However, the study by Mukong et al. [9] reveals that, while alcohol and tobacco alone contribute positively to income-related health inequality, the contribution from simultaneous exposure to these goods is enormous. This paper contributes to the growing literature by using more objective health outcomes to estimate the separate and joint effects and contribution of tobacco and alcohol use to income-related health inequality in Namibia. Using the 2015/16 Namibia Household Income and Expenditure Survey, we employ the Erreygers corrected concentration index and a decomposition technique to estimate the contribution of tobacco and alcohol use to income-related health inequality.

The paper first measures tobacco/alcohol-related health inequality and income-related health inequality for several health indicators, using the 2015/16 Namibia Household Income and Expenditure Survey and the Erreygers corrected concentration index. We estimated the contribution of tobacco and alcohol consumption to the income-related health inequality indices using the Wagstaff et al. [45] decomposition. The corrected concentration indices for tobacco use suggest that poor health is concentrated among tobacco users and even more among heavy users. The magnitude of the inequality is significantly higher when tobacco consumption intensity rather than tobacco prevalence is used. For example, the concentration indices for being sick and being diagnosed with a chronic disease are larger when tobacco prevalence is used than when tobacco intensity is used. However, these diseases are concentrated among non-alcohol users. The findings are similar to previous evidence on tobacco-related inequalities in health, which showed that the burden of ill health is significantly concentrated among individuals with high smoking intensity and longer smoking duration [9].

Two different measures of income are used to compute income-related health inequality using household per capita income and household per capita income by adult equivalent scale as measures of income. The results that some of the diseases are concentrated among the poor while some are common among the rich. The findings are generally consistent across the different measures of income. However, the magnitude of income-related inequality varies across the different measures of income and among health indicators. Vallejo-Torres and Morris [11] and Vallejo-Torres et al. [40] explore inequality trends in the United Kingdom (UK) and confirm a modest increase in income-related health inequality over time. Several other studies have confirmed that, while an amount of the related disease burden is concentrated among the poor, others are concentrated among the rich [9,10,57,58]. The pro-poor inequalities in some of the health outcomes may highlight the magnitude of lifestyle changes among those well-off [58]. Therefore, reducing the prevalence and intensity of tobacco use among the poor could be an effective policy for reducing the prevalence of lifestyle-related diseases and narrowing inequalities in health in Namibia.

Regarding the simultaneous consumption of tobacco and alcohol, the results suggest that consuming both goods contributes positively to income-related inequality in health. The contributions from tobacco consumption alone are generally positive while those from alcohol consumption alone are negative. Thus, individuals who jointly consume both goods have a higher risk of being diagnosed with a chronic disease than those who consume only one of these goods. This is consistent with existing literature suggesting that, while alcohol and tobacco alone are strongly related to the risk of ill health, simultaneous exposure to these goods has a stronger multiplicative effect on health [9,54,55,58].

## 5. Conclusions

Understanding the nature and key determinants of income-related health inequalities in a country with one of the highest levels of inequality in the world is important for designing and implementing appropriate policies aimed at tackling health disparities. The analysis controlled for covariates that are associated with health, so that the estimated effects of tobacco and alcohol consumption are unconditional. This paper has ascertained that a higher burden of many chronic diseases (ill health) is concentrated among the poor and this is further exacerbated by adopted lifestyles such as tobacco and alcohol use. Tobacco use contributes positively to income-related inequality in many chronic diseases, thereby widening the health inequality gap between the poor and the rich. On the contrary, alcohol consumption reduces (contributes negatively) to these health disparities. However, the simultaneous consumption of these goods has a strong multiplicative effect on income-related health inequality. While policy options for each of these goods could be essential in reducing their consumption and inequalities in health, there is a need to advocate additional measures that could simultaneously control their consumption. To fully understand the contribution of alcohol consumption to income-related health inequalities, future surveys in Namibia should consider collecting information on alcohol consumption intensity.

## Figures and Tables

**Table 1:** Mean health and differences in mean health by smoking and drinking status.

	Entire Sample	Smokers	Non Smokers	Mean Difference	Drinkers	Non Drinkers	Mean Difference
	Mean	Mean	Mean	Mean	Mean	Mean	Mean
Individual is sick	0.08	0.106	0.079	0.027 ***	0.082	0.083	−0.002
	(0.27)	(0.308)	(0.270)	(0.005)	(0.274)	(0.277)	(0.004)
Has a chronic disease	0.201	0.234	0.185	0.049 ***	0.178	0.200	−0.022 ***
	(0.401)	(0.423)	(0.388)	(0.007)	(0.383)	(0.400)	(0.005)
Individual is diabetic	0.020	0.016	0.017	−0.006	0.008	0.021	−0.013 ***
	(0.141)	(0.126)	(0.128)	(0.002)	(0.091)	(0.145)	(0.002)
Has high blood pressure	0.129	0.131	0.117	0.014 ***	0.108	0.125	−0.018 ***
	(0.335)	(0.337)	(0.321)	(0.006)	(0.310)	(0.331)	(0.004)
Has joint inflammation	0.022	0.043	0.021	0.022 ***	0.026	0.023	0.003 *
	(0.148)	(0.202)	(0.143)	(0.003)	(0.159)	(0.150)	(0.002)
Individual has cancer	0.004	0.003	0.003	0.000	0.003	0.004	−0.001 *
	(0.063)	(0.054)	(0.059)	(0.001)	(0.052)	(0.062)	(0.002)
Has heart disease	0.017	0.022	0.016	0.006 ***	0.013	0.019	−0.007 ***
	(0.127)	(0.147)	(0.126)	(0.002)	(0.113)	(0.138)	(0.002)
Individual is epileptic	0.008	0.010	0.008	0.002 *	0.007	0.009	−0.002 *
	(0.087)	(0.100)	(0.089)	(0.002)	(0.084)	(0.094)	(0.001)
Has respiratory disease	0.016	0.022	0.015	0.007 ***	0.012	0.018	−0.006 ***
	(0.126)	(0.146)	(0.120)	(0.002)	(0.110)	(0.132)	(0.002)
Has stomach ulcer	0.004	0.006	0.004	0.002 **	0.004	0.005	−0.001
	(0.066)	(0.079)	(0.066)	(0.001)	(0.066)	(0.070)	(0.001)
Has chronic kidney disease	0.004	0.006	0.003	0.003 ***	0.003	0.004	−0.001
	(0.064)	(0.078)	(0.056)	(0.001)	(0.056)	(0.062)	(0.001)
Individual is anaemic	0.003	0.002	0.003	−0.001	0.002	0.003	−0.001 *
	(0.055)	(0.046)	(0.053)	(0.001)	(0.046)	(0.055)	(0.001)
Has chronic psychological	0.006	0.012	0.007	0.004 ***	0.008	0.008	−0.001
	(0.079)	(0.107)	(0.085)	(0.002)	(0.087)	(0.090)	(0.001)
Has other chronic diseases	0.009	0.013	0.008	0.005 ***	0.009	0.009	0.000
	(0.094)	(0.114)	(0.088)	(0.002)	(0.092)	(0.093)	(0.001)

Note: Standard deviations/errors are in parentheses. *** Statistically significant at 1% level, ** statistically significant at 5% level and * statistically significant at 10% level.

**Table 2:** The distribution of disease burden by household income quintile.

Variables	Household Income per Capita					Household Income per Capita by Adult Equivalent				
	Poorer	Poor	Middle	Rich	Richest	Poorer	Poor	Middle	Rich	Richest
Individual consumes alcohol	17.81	20.39	19.64	20.31	21.85	18.23	20.51	20.30	20.09	20.87
Individual consumes tobacco	24.31	21.00	18.35	17.13	19.22	26.00	20.86	18.27	16.89	17.98
Individual is sick	24.33	23.57	20.98	17.26	13.86	23.72	23.43	20.93	18.72	13.20
Has a chronic disease	19.15	20.43	20.57	19.65	20.20	19.57	20.28	19.92	19.82	20.41
Individual is diabetic	7.73	14.04	20.37	21.78	36.07	8.43	12.88	20.37	22.25	36.07
Has high blood pressure	17.18	20.52	20.69	21.15	20.46	17.44	20.56	20.10	21.15	20.75
Has joint inflammation	20.45	20.62	23.05	21.10	14.77	21.59	19.81	22.24	20.62	15.75
Individual has cancer	13.79	14.94	22.99	11.49	36.78	14.94	14.94	18.39	13.79	37.93
Has heart disease	28.38	19.22	20.82	16.48	15.10	27.69	20.59	20.14	16.70	14.87
Individual is epileptic	27.36	32.08	18.87	11.79	9.91	28.30	30.19	21.23	10.85	9.43
Has respiratory disease	19.40	21.89	18.41	20.15	20.15	19.40	19.40	20.65	19.65	20.90
Has stomach ulcer	26.67	10.00	25.83	22.50	15.00	25.83	11.67	24.17	23.33	15.00
Has chronic kidney disease	26.88	19.35	17.20	21.51	15.05	25.81	20.43	16.13	20.43	17.20
Individual is anaemic	24.29	11.43	17.14	18.57	28.57	22.86	14.29	15.71	20.00	27.14
Has chronic psychological	32.20	24.39	21.95	14.15	7.32	33.66	24.88	20.00	14.15	7.32
Has other chronic diseases	14.48	14.48	19.00	16.29	35.75	16.29	14.48	14.93	20.36	33.94

**Table 3:** Income-related, tobacco-related and alcohol-related health inequality.

	(1)	(2)	(3)	(4)	(5)
Individual is sick	0.025 *	0.027 ***	0.009 ***	−0.037 ***	−0.035 ***
	(0.013)	(0.007)	(0.002)	(0.005)	(0.005)
Has a chronic disease	0.031 *	0.035 ***	−0.010 ***	0.025 ***	0.030 ***
	(0.018)	(0.003)	(0.004)	(0.007)	(0.007)
Individual is diabetic	0.007 ***	0.020 ***	−0.005 ***	0.022 ***	0.023 ***
	(0.006)	(0.003)	(0.001)	(0.002)	(0.002)
Has high blood pressure	0.022 ***	0.046 ***	−0.015 ***	0.031 ***	0.034 ***
	(0.014)	(0.008)	(0.003)	(0.006)	(0.006)
Has joint inflammation	−0.009	−0.005	0.003 **	−0.000	0.000
	(0.008)	(0.004)	(0.001)	(0.003)	(0.003)
Individual has cancer	0.004	−0.000	−0.001	0.003 ***	0.003 ***
	(0.003)	(0.001)	(0.001)	(0.001)	(0.001)
Has heart disease	−0.003	0.002	−0.002	−0.007 ***	−0.006 ***
	(0.006)	(0.003)	(0.001)	(0.002)	(0.002)
Individual is epileptic	−0.002	0.003	0.000	−0.008 ***	−0.008 ***
	(0.004)	(0.002)	(0.001)	(0.002)	(0.002)
Has respiratory disease	−0.004	0.005 *	−0.001	0.001	0.002
	(0.006)	(0.003)	(0.001)	(0.002)	(0.002)
Has stomach ulcer	0.004	0.003 *	0.001	−0.000	0.000
	(0.003)	(0.002)	(0.001)	(0.001)	(0.001)
Has chronic kidney disease	−0.005	0.002	0.000	−0.002 *	−0.002 *
	(0.004)	(0.002)	(0.001)	(0.001)	(0.001)
Individual is anaemic	0.001	0.001	−0.000	−0.000	−0.000
	(0.002)	(0.001)	(0.001)	(0.001)	(0.001)
Has chronic psychological	0.008 *	0.005 **	0.003 ***	−0.007 ***	−0.007 ***
	(0.004)	(0.002)	(0.001)	(0.001)	(0.001)
Has other chronic diseases	−0.006	0.006 **	0.000	0.008 ***	0.008 ***
	(0.005)	(0.002)	(0.001)	(0.002)	(0.002)

Note: Standard errors are in parentheses. The Erreygers corrected concentration indices (CCI) are presented in Columns (1) to (5). The CCI for tobacco consumers, tobacco consumption intensity, alcohol consumers, household per capita income and household per capita income by adult equivalent is presented in Columns (1) to (5), respectively. *** Statistically significant at 1% level, ** statistically significant at 5% level and * statistically significant at 10% level.

**Table 4:** Regression estimates for tobacco/alcohol-related health outcomes.

	(1)	(2)	(3)	(4)	(5)	(6)
Consumes alcohol	0.013 ***	0.023 ***	0.005 ***			
	(0.005)	(0.006)	(0.001)			
Consumes tobacco	0.009 ***	0.012	0.004 *			
	(0.002)	(0.012)	(0.002)			
Interaction between tobacco and alcohol	0.029 **	0.012 **	0.005			
	(0.012)	(0.006)	(0.003)			
Consumes tobacco and alcohol				0.018 ***	0.023 **	0.006 ***
				(0.007)	(0.010)	(0.002)
Consumes tobacco only				0.002	0.012	0.004 *
				(0.009)	(0.012)	(0.002)
Consumes alcohol only				0.013 ***	0.023 ***	0.006 ***
				(0.005)	(0.006)	(0.001)
Constant			0.019 ***			0.019 ***
			(0.004)			(0.004)
Observations	17,813	17,813	17,813	17,813	17,813	17,813
R-squared			0.180			0.180

Notes: Results in Columns (1) and (4) are probit estimates for being sick, Columns (2) and (5) are probit estimates for having a chronic disease and Columns (3) and (6) are OLS estimates for the health index of all chronic diseases. The analysis control household income, place of residence, education, age, gender, marital status and employment. Standard errors in parentheses, *** *p* < 0.01, ** *p* < 0.05, * *p* < 0.1.

**Table 5:** The contribution of smoking and alcohol to income-related inequalities.

	Alcohol Consumption				Tobacco Consumption			
	Elasticity	CI	Contr.	%	Elasticity	CI	Contr.	%
Individual is sick	−0.001	0.011	−0.000	−0.527	0.000	−0.018	−0.000	0.063
Individual has a chronic disease	−0.015	0.011	−0.001	−5.982	0.002	−0.018	−0.000	0.595
Individual is diabetic	−0.004	0.011	−0.000	−1.514	0.000	−0.018	−0.000	0.025
Individual has high blood pressure	−0.013	0.011	−0.001	−5.100	−0.002	−0.018	0.000	−0.639
Individual has joint inflammation	−0.000	0.011	−0.000	−0.076	0.001	−0.017	−0.000	0.416
Individual has cancer	−0.001	0.011	−0.000	−0.284	0.000	−0.018	−0.000	0.050
Individual has heart disease	−0.002	0.011	−0.000	−0.950	−0.000	−0.018	0.000	−0.019
Individual is epileptic	−0.001	0.011	−0.000	−0.543	0.000	−0.018	−0.000	0.164
Individual has a respiratory disease	−0.001	0.011	−0.000	−0.313	0.001	−0.018	−0.000	0.397
Individual has a stomach ulcer	−0.000	0.011	−0.000	−0.040	0.000	−0.018	−0.000	0.150
Individual has chronic kidney disease	0.001	0.011	0.000	0.279	−0.000	−0.018	0.000	−0.046
Individual is anaemic	−0.000	0.011	−0.000	−0.159	0.000	−0.018	−0.000	0.051
Individual has chronic psychological	−0.001	0.011	−0.000	−0.325	0.000	−0.018	−0.000	0.021
Has other chronic diseases	−0.000	0.011	−0.000	−0.130	0.000	−0.018	−0.000	0.020
Health index	−0.003	0.011	−0.000	−1.261	0.000	−0.018	−0.000	0.099

Note: Results presented in this table are elasticities, Erreygers corrected concentration index, contributions and percentage contributions of alcohol and tobacco consumption to income-related health inequality. The results are obtained by decomposition of the income-related health inequality indices into health-related covariates, including tobacco and alcohol use. The health indicators are all binary outcomes equivalent to 1 if the respondent is diagnosed with a given disease. The health index is continuous with high values representing poor health outcomes. The tobacco and alcohol consumption variables are both binaries equal to 1 if the respondent is a current smoker or drinker regularly. Other variables were also controlled including residential type, education, categories for age, gender, marital status and employment.

**Table 6:** The combined contribution of tobacco and alcohol consumption to income-related inequalities in health.

	Health Index				Individual Is Sick				Individual Has a Chronic Disease			
	Elasticity	CI	Contribution	%	Elasticity	CI	Contribution	%	Elasticity	CI	Contribution	%
Individuals consume tobacco and alcohol	0.001	0.022	0.000	0.245	0.0003	−0.022	0.000	0.129	0.003	0.022	0.000	1.025
Individuals consume tobacco only	0.000	0.004	0.000	0.066	0.0006	0.004	0.000	0.249	0.001	0.004	0.000	0.292
Individuals consume alcohol only	−0.002	0.033	−0.000	−0.922	−0.0020	0.033	−0.000	−0.802	−0.011	0.033	−0.002	−4.464

Note: Results presented in this table are elasticities, Erreygers corrected concentration index, contributions and percentage contributions of household per capita income, alcohol and tobacco consumption to income-related health inequality. The results are obtained by decomposition of the income-related health inequality indices into health-related covariates, including tobacco and alcohol use. The sick and those with chronic disease variables are binary and the health index is continuous, with high values representing poor health outcomes. The tobacco and alcohol use variables are categorical. Other variables were also controlled including household per capita income, residential type, education, categories for age, gender, marital status and employment.

## Data Availability

The datasets used for the analysis in this paper were received from the Namibia Statistics Agency. They are available from the corresponding author upon reasonable request.
